# Interactive effects of electrical conductivity and light intensity on growth, yield, and nutrient dynamics of hydroponic lettuce

**DOI:** 10.1038/s41598-026-44508-2

**Published:** 2026-03-24

**Authors:** Nazmin Akter, Laura Cammarisano, Md Shamim Ahamed

**Affiliations:** 1 Department of Plant Sciences, University of California, Davis, USA; 2 Department of Biological and Agricultural Engineering, University of California, Davis, USA

**Keywords:** Lactuca sativa, Electrical conductivity, Light intensity, Nitrate accumulation, Mineral uptake, Vertical farming, Ecology, Ecology, Environmental sciences, Plant sciences

## Abstract

Abstract Precise management of nutrient solution properties, such as electrical conductivity (EC), and environmental factors, such as light intensity (LI), is essential for optimizing crop yield and quality in hydroponic production. This study evaluated the individual and combined effects of two EC ranges (EC1: 1.5–2.0 dS m-1; EC2: 4.5–6.0 dS m-1) and three LI levels (L1 = 145, L2 = 185, and L3 = 240 $\mu$mol m-2 s-1) on the growth, yield, leaf mineral uptake, and nitrate accumulation of butterhead lettuce grown in hydroponic system under artificial lighting. The greatest leaf area (1338.31 cm2) and yield (57.97 g plant-1) were observed in EC1L3 treatment, corresponding to reductions of 75% and 77.2%, respectively, compared to EC2L3 (330.79 cm2 and 13.98 g plant-1), indicating the adverse effects of salinity stress. Furthermore, within the same EC1 level, LI positively impacted yield, which increased by 47% under EC1L3 compared to EC1L1. Mineral composition analysis revealed that EC2 significantly reduced the uptake of essential macronutrients nitrogen (N), phosphorus (P), potassium (K), calcium (Ca), magnesium (Mg), and sulfur (S) and micronutrients boron (B), zinc (Zn), manganese (Mn), iron (Fe), and copper (Cu), whereas LI had no significant effect, except for a minor interaction in B uptake. An inverse correlation ($R^2$= -0.80) between solution and leaf nitrate levels was observed; however, leaf nitrate content was low and did not differ significantly across treatments. This closed-loop hydroponic study in a vertical cultivation setup offers practical insights for efficient controlled environment agriculture (CEA) systems. The results highlight that the interaction between EC and LI plays a significant role in influencing lettuce morphology and yield, but not mineral uptake. Maintaining EC between 1.5 and 2.0 dS m-1 and LI at 240 µmol m-2 s-1 resulted in the highest growth and yield under the tested conditions. The findings from this study will be critical for future research on investigating the interactive effect of different light and nutrient recipes for other hydroponic crops.

## Introduction

Lettuce (*Lactuca sativa* L. var. capitata, cv. Higgs) is a widely cultivated leafy green vegetable grown hydroponically and eaten worldwide for its crisp texture, mild flavor, and health-promoting properties. It is a good source of dietary fiber, vitamins A and K, and contains bioactive compounds such as antioxidants and phenolics^1,2^. As a salad and frequent sandwich topper, lettuce is the most commonly used leafy green vegetable in the United States. In 2023, it ranked as California’s 4^th^ most valuable commodity, generating over $3.93 billion in farm cash receipts^3,4^. The growing demand for fresh, nutritious vegetables has driven the development of sustainable production technologies such as hydroponics and vertical farming^5,6^. Hydroponic production has the potential to regulate growth conditions with great precision, resulting in improved productivity, reduced water and nutrient use, and improved crop quality^7–9^. Hydroponics, known as soilless production, is commonly practiced in greenhouses and indoor vertical farming systems. Hydroponic production is 75–95% water efficient because of closed-loop operation and nutrient-efficient because of the precision of nutrient treatment flexibility^10^. However, a key concern with hydroponically grown leafy greens is the optimal supply of nutrients and light, as improper management can lead to the accumulation of excess nitrate in edible tissues. This is largely due to the complexity of nutrient dosing, particularly since most commercial fertilizers contain significant amounts of nitrogen. Although a vital nutrient, lettuce is particularly susceptible to nitrate accumulation, which can be toxic in excess^11^. The European Commission Regulation No. 1258/2011 calls for the maximum allowed nitrate content in lettuce to be 4000 mg/kg fresh weight (FW) for summer crops and 5000 mg/kg FW for winter crops grown under greenhouses. Any excess amount will result in methemoglobinemia, also known as “blue baby syndrome” in infants, and the potential formation of carcinogenic nitrosamines in adults^12,13^.

Nitrate uptake in lettuce is modulated by various environmental factors, including electrical conductivity (EC) of the nutrient solution and light intensity (LI), both of which affect not only nitrate metabolism but also overall plant growth, yield, mineral composition, and quality. EC is an extremely crucial parameter that reflects the total ion content in the nutrient solution. EC directly affects the osmotic status around the roots, leading to water and nutrient uptake. Optimal levels of EC promote balanced nutrient uptake and physiological functions, whereas extremes lead to deficiency or osmotic stress^7,14^. Several studies^15–17^ have indicated that increased EC levels reduce the efficiency of nitrate uptake as well as inhibit nutrient transport functions. This can result in altered mineral composition, stunted growth, and increased stress reaction^16,18^. Therefore, maintaining an optimal EC range is vital for balancing growth and nitrate content in lettuce. Also, LI controls nitrate dynamics as it can affect photosynthesis, plant morphology, and secondary metabolite production in lettuce^19–21^. LI is directly linked to the induction of nitrate reductase enzymes required to convert nitrate into amino acids. In high-light conditions, enhanced photosynthesis generates more energy and electron carriers (such as NADPH), which support increased nitrate reduction and assimilation^22^. Several studies^17,22–24^ have shown that high light intensity increases plant biomass, decreases nitrate content in leaves, and improves crop quality attributes such as antioxidant levels, pigmentation, and texture through metabolic conversion.

In general, the individual effects of EC and LI on lettuce growth, quality, and nitrate accumulation have been well-documented. For example^6^, evaluated five EC levels (0.5, 0.7, 0.9, 1.2, and 2.0 dS m^−1^) in an ebb and flow hydroponic system and found that lettuce growth was optimal at 0.9 and 1.2 dS m^−1^, while chlorophyll and nitrogen concentrations were highest at 2.0 dS m^−1^. Conversely^18^, observed that high EC levels (3.6–5.6 dS m^−1^) disrupted water and ion balance in lettuce, leading to reduced yield. Similarly^15^, found that increasing EC from 0.65 to 0.9 dS m^−1^led to greater nitrate accumulation and faster growth in greenhouse-grown lettuce. Also^16^, noted that different lettuce types experienced stress and slower growth when EC levels reached 10 dS m^−1^. Even^25^ reported that high EC induced by salinity (10–12 dS m^−1^) significantly reduced lettuce biomass and photosynthetic performance. Regarding LI^23^, demonstrated that applying a short-term pre-harvest treatment with increased LI (PPFD; from 250 to 350 $\mu \textrm{mol}\,\textrm{m}^{-2}\,\textrm{s}^{-1}$) combined with a 50% reduction in nitrogen supply significantly enhanced nitrate assimilation and reduced nitrate accumulation in lettuce leaves by stimulating nitrate reductase activity. And^5^, found that combining moderate to high light intensities (250–350 $\mu \textrm{mol}\,\textrm{m}^{-2}\,\textrm{s}^{-1}$) with reduced nitrogen supply (1/4–3/4 nutrient solution concentration; NSC) improved lettuce growth, nutrient content, and antioxidant levels, while significantly reducing nitrate accumulation, especially under 350 $\mu \textrm{mol}\,\textrm{m}^{-2}\,\textrm{s}^{-1}$ $\times$ 1/4 NSC treatment, which was identified as optimal for nutritional quality in plant factory conditions. Therefore, it’s critical to investigate the interactive impact of light and nutrient supply for optimal growth.

There is little information on how EC and LI influence lettuce physiological processes and nitrate assimilation in hydroponic production. Few studies^26^have investigated interactive effects on lettuce growth in plant factories, but they have not addressed nitrate accumulation in vertical farming systems. Notably^5^, examined the combined effects of light intensity and nutrient solution concentration on lettuce nutritional quality and antioxidant properties. In their study, nutrient solution dilution levels were varied, which in turn altered the EC of the solution. In a related study^27^, explored the interactive effects of salinity and light intensity on kale performance in indoor hydroponics, highlighting the importance of optimizing both factors for sustainable crop production under stress conditions. Given the increased emphasis on food security and nutrient-dense crop production, especially in hydroponic farming, an integrated understanding of nitrate dynamics is essential. To address this knowledge gap, the study aimed to investigate the effects of EC and LI on the growth, yield, mineral composition, and nitrate accumulation of hydroponic lettuce grown in an indoor farming system under artificial lighting. It was hypothesized that 1) plant morphology and yield would benefit from increased LI but not from elevated EC; 2) a significant interaction effect between EC and LI would diminish the benefits of increased LI and potentially increase nitrate accumulation.

## Materials and methods

The experiment was conducted in a three-tier climate-controlled reach-in chamber unit in the Controlled Environment Facilities at the University of California, Davis, from December 2024 to March 2025.

### Description of seedlings preparation

Lettuce (*Lactuca sativa* L. var. capitata, cv. Higgs) pelleted seeds (Johnny’s Selected Seeds, Winslow, Maine, USA) were germinated in organic plugs (Ventana Plant Science, USA). ‘Higgs’ is a butterhead lettuce cultivar characterized by a compact growth habit and uniform head development, and it is commonly used in hydroponic and CEA systems. Each plug measured 3.2 $\times$ 3.2 cm and was a mixture of coco coir and peat moss (with OMRI-listed ingredients and *Trichoderma*). After incubation at 21$^\circ$C and in dark conditions for two days, the plugs were subjected to a controlled climate to allow homogeneous plant growth for 13 days. The set point temperature of the growth chamber was 23/18$^\circ$C (day/night). The average measured temperature was 21.00 ± 0.04$^\circ$C. The set point relative humidity was 75%, and the measured average RH was 81.0 ± 0.1%. The climate conditions within the treatment chamber were monitored and recorded every 30 minutes throughout the experiment using a Guardian CEA Multi-Sensor Monitor (Model SM-500, Apogee Instruments, Logan, UT, USA). The CO$_2$ concentration was not controlled, and ambient CO$_2$ levels were assumed in the chamber because of ventilated air circulation. The photoperiod was set to 16/8 h (day/night), and the light was provided by an LED lamp (Spider Farmer, G series, China) placed 20 cm above the cultivation surface. Light intensity was measured at 15 cm below the lamp and 5 cm above the cultivation surface; the average Photon Flux Density was 154.95 ± 4.66 $\mu$mol m^−2^ s^−1^ (LI-180 spectrometer, LI-COR Biosciences, Lincoln, Nebraska, United States). The nutrient solution was prepared once, maintaining an EC of 1.50 dS m^–1^ and a pH of 6.5. Plants were manually irrigated with the prepared nutrient solution twice a day.

### Description experimental setup and growing conditions

**Fig. 1:**
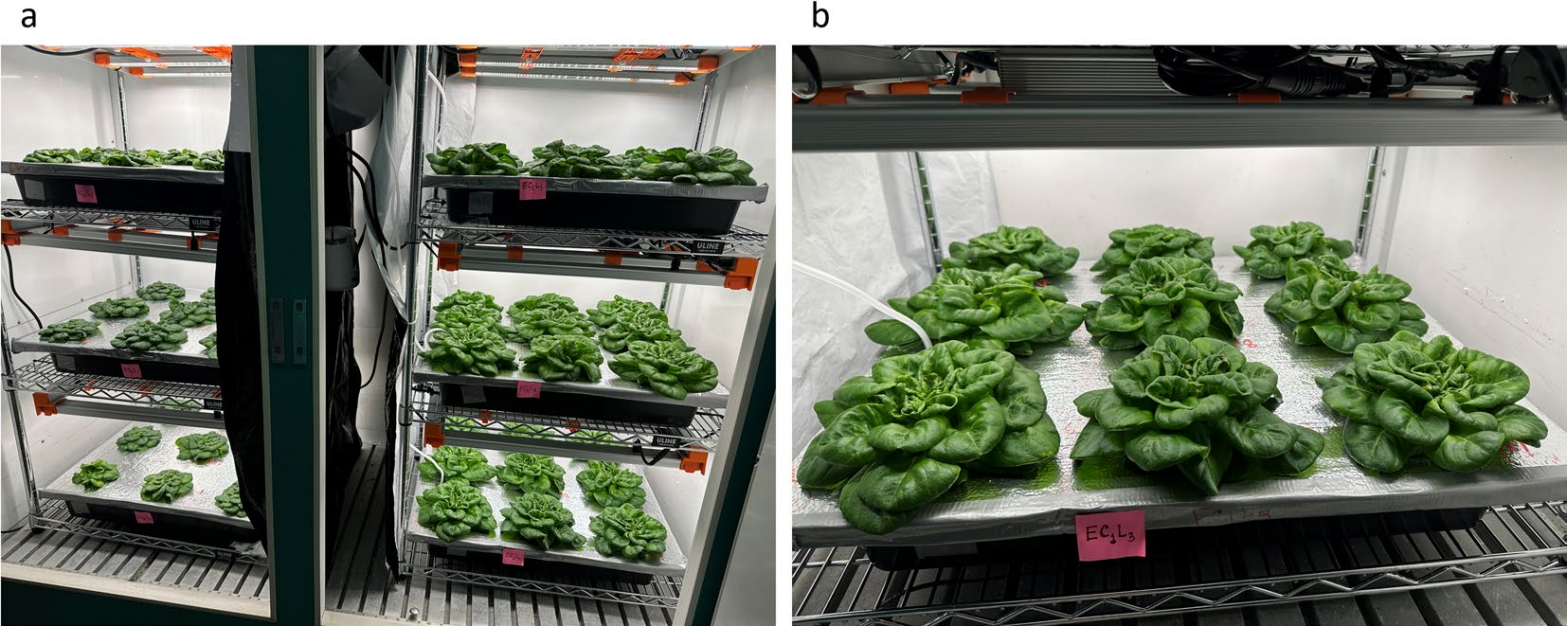
Images showing the experimental setups within the reach-in climate-controlled growth chamber (a), each layer hosted 9 plant replicates (b) and represented one of the six treatments.

**Fig. 2:**
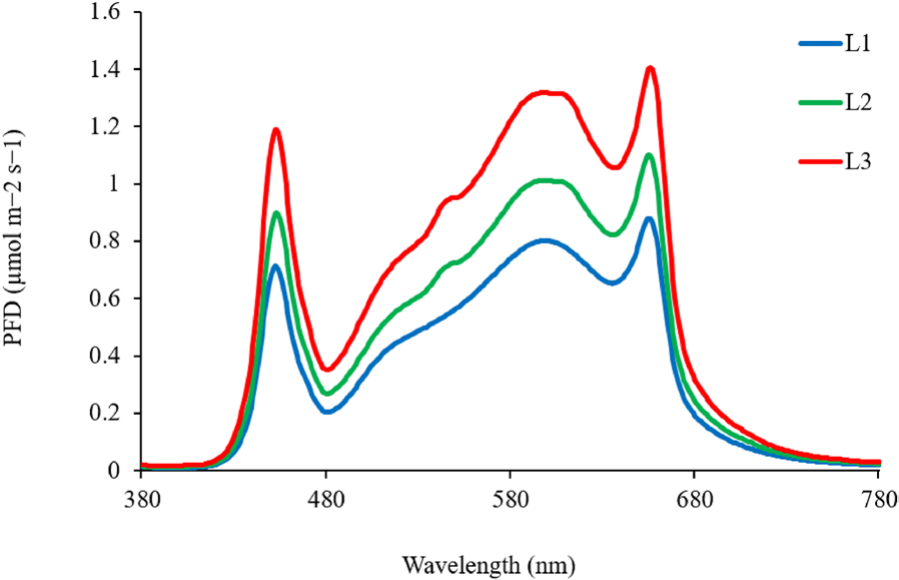
Light spectral distribution of the three light intensity treatments.

The growing system consisted of two multi-layer racks within the reach-in chamber, each layer equipped with a dimmable broadband white LED lamp (Spider Farmer, G series) and a deep-water hydroponic box (60.96 cm $\times$ 60.96 cm $\times$ 7.5 cm), forming six growth areas (Fig.1a), each representing a unique treatment combination. Two EC levels (EC1 and EC2) and three LI levels (L1, L2, L3) were tested (Table1). These EC and LI levels were selected to represent commonly used and elevated conditions in hydroponic lettuce production, allowing evaluation of plant growth and nutrient responses under practical controlled environment conditions^16,23^. Treatments were applied for 21 days, starting 15 days after sowing, including 2 days of dark treatment. Seedlings with six leaves were transferred to a treatment setup in the same growth chamber. Treatments were randomized within two temporal replications. Each hydroponic box accommodated nine seedlings on a Styrofoam plate at a planting density of 24 plants m^–2^ (Fig.1b). A water flow pump was used in each box to ensure root aeration. Nutrient solution parameters (EC, pH) were monitored daily (GroLine pH/EC/TDS meter, Hanna Instruments, USA). EC was adjusted using nutrient solutions A and B (Humboldt’s Secret Base A and B Bundle, San Diego, CA, USA), and pH was adjusted using pH Up (KOH solution, Humboldt’s Secret). One side of the growing system with three layers was maintained at EC1 (1.5–2.0 dS m^–1^) and the other side at EC2 (4.5–6.0 dS m^–1^). Lamps were set 20 cm from the cultivation surface and dimmed to achieve target photosynthetically active photon flux density (PPFDs) of 145, 185, and 240 $\mu \textrm{mol}\,\textrm{m}^{-2}\,\textrm{s}^{-1}$ for LI treatments. Light intensity was measured using the LI-180 spectrometer at 12 evenly spaced points, 5 cm above the cultivation plate. Average PPFDs and standard errors are reported as mean ± SE (Table 2). Spectral distribution of treatments was consistent across intensities (Fig.2) as per recommended by the most popular McCree Curve^28^.

**Table 1:** Overview of light intensity (LI) and electrical conductivity (EC) treatments applied in the experiment.

**Light treatments**	**LI **($\mu \textrm{mol}\,\textrm{m}^{-2}\,\textrm{s}^{-1}$)	**EC treatments**	**EC **(**dSm**^−1^)
L1	145.0	EC1	1.5–2.0
L2	185.0	EC2	4.5–6.0
L3	240.0

**Table 2:** Measured photon flux density (PFD), photosynthetic photon flux density (PPFD), daily light integral (DLI), electrical conductivity (EC), and pH of nutrient solutions under different EC and LI treatment combinations in a vertical hydroponic lettuce system.

**Treatment**	**PFD**	**PPFD**	**DLI**	**EC**	**pH**
	($\mu \textrm{mol}\,\textrm{m}^{-2}\,\textrm{s}^{-1}$)	($\mu \textrm{mol}\,\textrm{m}^{-2}\,\textrm{s}^{-1}$)	(mol m^−2^ d^−1^)	(dSm^−1^)	
EC1L1	144.00 ± 1.1	140.00 ± 1.2	8.06	1.98 ± 0.02	6.02 ± 0.02
EC1L2	185.00 ± 0.0	180.00 ± 0.0	10.37	1.95 ± 0.01	6.07 ± 0.02
EC1L3	236.00 ± 5.2	229.00 ± 5.0	13.19	1.95 ± 0.04	6.00 ± 0.04
EC2L1	145.00 ± 0.8	141.00 ± 0.7	8.12	5.40 ± 0.08	4.90 ± 0.07
EC2L2	188.00 ± 2.3	182.00 ± 2.2	10.48	5.60 ± 0.03	4.90 ± 0.20
EC2L3	239.00 ± 0.9	232.00 ± 0.8	13.36	5.40 ± 0.09	4.80 ± 0.20

Values represent means ± standard error (SE) based on two measurements taken throughout two temporal replications.

### Performance metrics

In this study, each treatment was replicated twice, with nine plants per replication. Three randomly selected plants from each treatment were considered for morphological and yield measurement, and the remaining six plants were sampled for pigment and nutrient composition analysis. The performance of the treatment was evaluated based on morphology, yield, and nutrient composition.

####  Morphological parameters and yield

Morphological measurements were conducted three weeks after transplanting, following a destructive harvest. Various parameters, such as leaf number, canopy area, and total leaf area, were evaluated. The canopy and leaf area of three lettuce plants per light treatment were quantified by capturing digital photographs of the whole plant head (from above) and the individual leaves. Canopy or rosette area images were taken from a distance of 30 cm above the plant on a white flat surface under ambient room light. Individual leaves were then detached, counted, and placed on a white flat surface to capture their leaf area images from a distance of 56 cm using an iPhone 14 Pro camera. The images were analyzed using ImageJ software (V1.8.0; National Institute of Health and the Laboratory for Optical and Computational Instrumentation, USA) (supplementary file). After imaging, FWs of the same three plants were measured using an analytical balance (OHAUS, CR 621, China) at final harvest. The leaves were subsequently dried in an oven (Shel Lab, General Purpose Incubator, Sheldon Manufacturing Inc., USA) at 70$^\circ$C for 72 hours to determine dry weight. Root length was measured in centimeters using a measuring ruler.

The specific leaf area (SLA) and dry matter content (DMC) were calculated as follows:

1 $$\begin{aligned} & \text {SLA} = \frac{\text {Leaf area}}{\text {Leaf dry weight}} \end{aligned}$$

2 $$\begin{aligned} & \text {DMC} = \left( \frac{\text {Dry weight}}{\text {Fresh weight}} \right) \times 100 \end{aligned}$$

#### Pigments and nutrients quantification

##### Chlorophyll Content Index (CCI)

Before the final harvest, 35 days after sowing, three leaves from five randomly selected plants in each light treatment were chosen for chlorophyll measurements. A portable CCM-200 (Opti-Sciences, Hudson, New Hampshire, United States) was used to determine the CCI nondestructively. Leaves selected for measurement were generally from the third layer above the base of the plant, fully expanded, and uniform in size. To ensure consistency, measurements were taken from the middle region of the adaxial side of the leaf blade^29^.

##### Total nitrogen

After the 36-day growth period, six whole plant samples, randomly selected from each treatment, were chosen for chemical analysis. After harvest, the plants were immediately immersed in liquid nitrogen and stored at −80$^\circ$C. Later, the samples were dried in an oven at 60 ± 5 $^\circ$ C. The dried tissue was then ground into a fine powder using a 40-mesh screen and stored in clean containers for nitrate and mineral quantification. The total nitrogen content in lettuce tissue was assessed using a combustion method. Three grams of dried and ground samples were combusted in an induction furnace, and the resulting gases were analyzed using thermal conductivity and infrared detectors^30^. This technique converts all nitrogen compounds into detectable gases, allowing for accurate quantification. The detection limit for nitrogen was about 0.02% as recommended by^31^.

##### Mineral composition

About 5 grams of dried, ground tissue were digested in sealed containers using nitric acid and hydrogen peroxide in a microwave to analyze mineral nutrients in lettuce. This technique guarantees complete dissolution of plant tissues while reducing contamination and minimizing the loss of volatile elements. The resulting solutions were examined through Inductively Coupled Plasma Atomic Emission Spectrometry (ICP-AES) at the UC Davis analytical lab, which allows for high sensitivity and accurate simultaneous detection of multiple elements. The procedures for digestion and analysis followed the protocols outlined by^32,33^.

##### Nitrate-nitrogen (${\textrm{NO}_{3}-\textrm{N}}$) in dry leaf

Soluble nitrate-nitrogen (${\textrm{NO}_{3}-\textrm{N}}$) was measured in lettuce tissue using a 2% acetic acid extraction method, followed by Flow Injection Analysis (FIA). About 3 grams of dried, ground plant material was extracted, and the solution was passed through a copperized cadmium column to convert nitrate into nitrite. The nitrite reacted with sulfanilamide and coupled with *N*-(1-naphthyl) ethylenediamine dihydrochloride, resulting in a diazo compound, which was measured for absorbance at 520 nm. This procedure was adapted from the methods outlined by^34,35^.

##### Nitrate-nitrogen (${\textrm{NO}_{3}-\textrm{N}}$) of nutrient solution

Nitrate content in the hydroponic nutrient solution was measured at three distinct intervals across the entire experimental period using a Nutrient Analyzer Photometer following the manufacturer’s protocol. A 10 mL sample was diluted to 100 mL with deionized water, and then further diluted by a factor of two. A 10 mL sample from this dilution was reacted with the nitrate reagent per protocol. After the required reaction time, the sample was placed in the calibrated photometer, and the nitrate concentration was recorded in mg/L. Measurements were repeated to track variations over time, following the HANNA manual (HI83325, Hanna Instruments, USA), by^36^.

###  Statistical analysis

Statistical analyses were performed using the R language program (version 22.1.0.195; VSN International Ltd, Hemel Hempstead, UK) with the following packages: agricolae, car, ggplot2^37–39^,. Two-way ANOVA was performed, and the Least Significant Difference (LSD) test was used for mean separation at a significance level of $\alpha = 0.05$. Homogeneity of variances was assessed using a scale location plot, and normality of residuals was evaluated using a QQ plot. Data were presented as mean ± standard error of the mean based on common variance. For mineral composition analysis, when the treatment interaction was not statistically significant, a one-way ANOVA was conducted. Additionally, regression analysis was used to evaluate the correlation between ${\textrm{NO}_{3}-\textrm{N}}$ concentrations in the nutrient solution and leaf tissue. All measurements were statistically replicated twice (two temporal replications of the experiment).

## Results

### Effect of LI and EC on morphology and growth

**Fig. 3:**
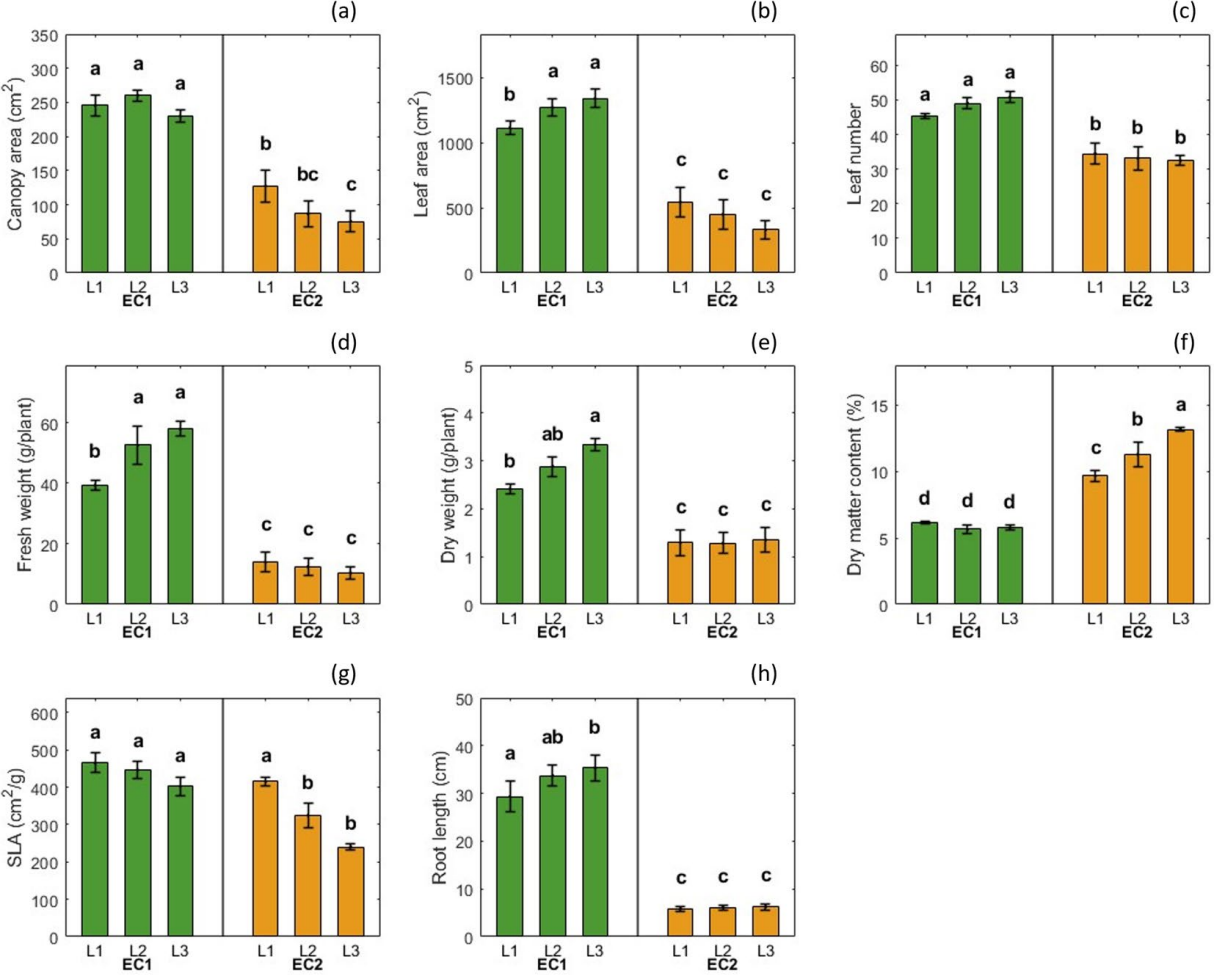
Effects of Electrical Conductivity (EC1 = 1.5–2.0 dS m^−1^, EC2 = 4.5–6.0 dS m^−1^) and Light Intensity (L1 = 145 $\mu \textrm{mol}\,\textrm{m}^{-2}\,\textrm{s}^{-1}$, L2 = 185 $\mu \textrm{mol}\,\textrm{m}^{-2}\,\textrm{s}^{-1}$, L3 = 240 $\mu \textrm{mol}\,\textrm{m}^{-2}\,\textrm{s}^{-1}$) on canopy area (a), leaf area (b), leaf number (c), fresh weight (d), dry weight (e), dry matter content (f), specific leaf area (g), and root length (h) of hydroponically grown lettuce in a vertical farming system. Values represent the mean ± standard error (SE) of two temporal replicates (independent experimental runs) per treatment. Different letters above bars indicate significant differences at $P \le 0.05$.

Canopy area was significantly affected by EC ($P \le 0.05$), whereas neither LI ($P = 0.12$) nor the EC $\times$ LI interaction ($P = 0.23$) showed a significant effect. Under EC1, canopy area remained high and statistically similar across all light levels, ranging from 230.05 cm^2^ at L3 to 260.31 cm^2^ at L2. In contrast, under EC2, canopy area decreased significantly with increasing LI, from 127.26 cm^2^ at L1 to 75.69 cm^2^ at L3. Overall, EC2 treatments exhibited significantly smaller canopy areas than EC1, with the lowest values observed at L2 and L3 (Fig.3a). Leaf area was significantly affected by EC ($P \le 0.05$) and by the EC $\times$ LI interaction ($P \le 0.05$), whereas LI alone had no significant effect ($P = 0.92$). Under EC1, leaf area increased with increasing LI, rising from 1113.70 at L1 to 1270.50 at L2 and 1338.31 at L3, with L2 and L3 being statistically similar and significantly greater than L1. In contrast, under EC2, leaf area was substantially reduced and remained low across all LI levels, ranging from 543.17 at L1 to 330.79 at L3, with no significant differences among L1–L3. Overall, leaf area under EC2 was significantly lower than under EC1. Leaf number was significantly affected by EC ($P \le 0.05$), whereas LI ($P = 0.72$) and the EC $\times$ LI interaction ($P = 0.23$) showed no significant effects. Plants grown under EC1 consistently produced greater leaf numbers across all LI levels, ranging from approximately 45 to 50 leaves per plant. In contrast, plants under EC2 exhibited significantly fewer leaves, with values ranging from 32 to 34 leaves per plant (Fig.3c). In terms of yield, FW was significantly affected by EC ($P \le 0.05$) and by the EC $\times$ LI interaction ($P \le 0.01$), while LI alone had no significant main effect ($P = 0.09$). Under EC1, FW increased with increasing LI, from approximately 39 g plant^–1^ at L1 to 52.6 g plant^–1^ at L2 and 58.0 g plant^–1^ at L3, with L2 and L3 producing significantly greater FW than L1. In contrast, under EC2, FW remained consistently low across all LI levels, ranging from approximately 10 to 14 g plant^–1^, with no significant differences among LI treatments (Fig. 3d). Dry weight was significantly affected by EC ($P \le 0.05$), whereas LI ($P = 0.06$) and the EC $\times$ LI interaction ($P = 0.12$) showed no significant effects. Under EC1, dry weight increased from 2.41 g plant^–1^ at L1 to 3.34 g plant^–1^ at L3, with L3 being significantly greater than L1, while L2 was statistically similar to both. In contrast, dry weight under EC2 remained consistently low across all LI levels, ranging from 1.28 to 1.35 g plant^–1^ (Fig. 3e).

Dry matter content (DMC) was significantly affected by EC, LI, and their interaction (all $P \le 0.05$). Under EC2, DMC increased with LI, from 9.65% at L1 to 13.16% at L3. All values under EC2 were significantly greater than those under EC1. In contrast, under EC1, DMC remained low and relatively unchanged across LI levels (5.65% to 6.14%) with no significant differences among L1–L3 (Fig. 3f). Specific leaf area (SLA) was significantly affected by EC and LI (both $P \le 0.05$), whereas the EC $\times$ LI interaction showed a marginal effect ($P = 0.051$). Under EC1, SLA remained high and statistically similar across all LI levels, ranging from 464.95 to 402.68 cm^2^ g^–1^. In contrast, under EC2, SLA decreased significantly with increasing LI, from 415.32 cm^2^ g^–1^ at L1 to 239.61 cm^2^ g^–1^ at L3 (Fig. 3g). Root length was significantly affected by EC ($P \le 0.05$), whereas LI ($P = 0.25$) and the EC $\times$ LI interaction ($P = 0.34$) showed no significant effects. Under EC1, root length increased with increasing LI, from 29.33 cm at L1 to 35.33 cm at L3, with L3 being significantly greater than L1. In contrast, root length under EC2 remained consistently low across all LI levels, ranging from 5.88 to 6.27 cm, with no significant differences among treatments (Fig. 3h).

Overall, the morphological metrics, including leaf number, leaf area, fresh, and dry weights, were consistently lower under EC2 than EC1, with statistically significant differences across all metrics.

### Effect of LI and EC on plant pigments and nitrate accumulation

**Fig. 4:**
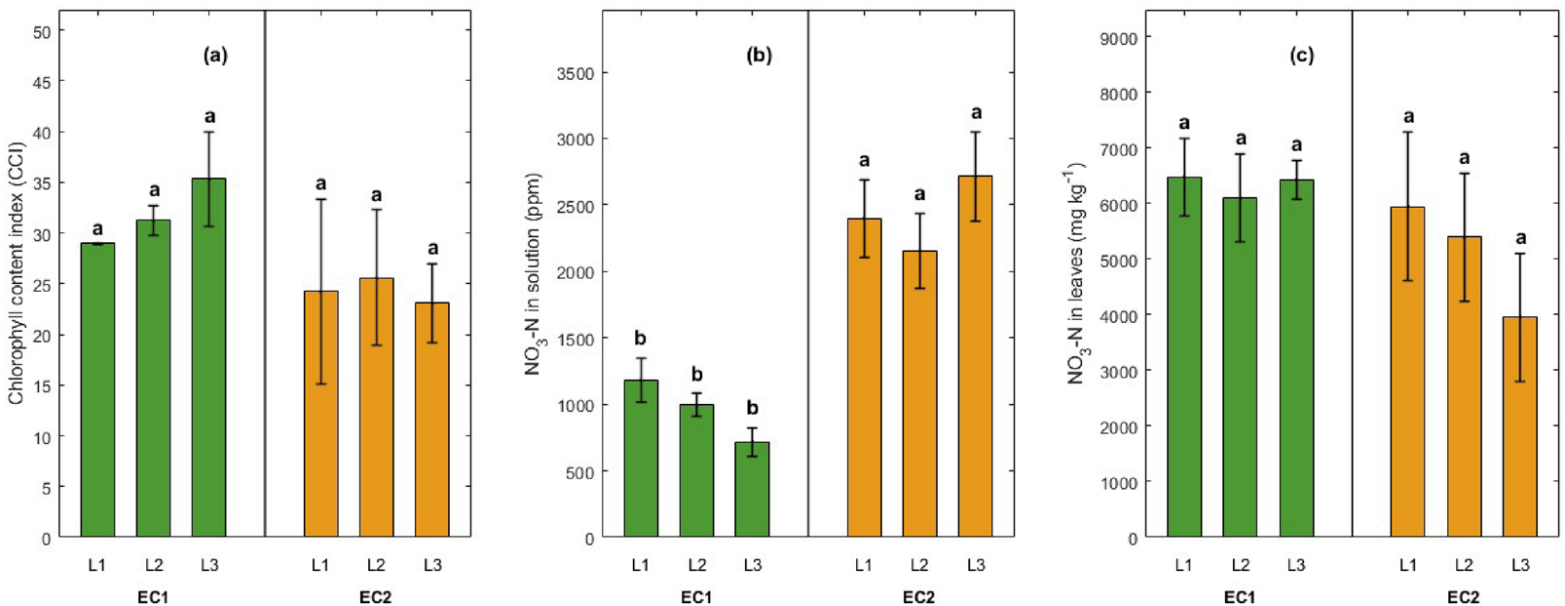
Effects of Electrial Conductivity (EC1 = 1.5–2.0 dS m^−1^, EC2 = 4.5–6.0 dS m^−1^) and Light Intensity (L1 = 145 $\mu \textrm{mol}\,\textrm{m}^{-2}\,\textrm{s}^{-1}$, L2 = 185 $\mu \textrm{mol}\,\textrm{m}^{-2}\,\textrm{s}^{-1}$, L3 = 240 $\mu \textrm{mol}\,\textrm{m}^{-2}\,\textrm{s}^{-1}$) on (a) chlorophyll content, (b) NO_3_-N concentration in the nutrient solution, and (c) NO_3_-N accumulation in lettuce leaves. Bars represent means ± standard errors from two independent experimental replicates. Different letters above bars indicate statistically significant differences among treatments at $P \le 0.05$.

CCI was not significantly affected by EC ($P = 0.13$), LI ($P = 0.88$), or their interaction ($P = 0.75$) (Fig.4a). NO$_3^-$-N concentration in the nutrient solution was significantly affected by EC ($P \le 0.05$), whereas LI ($P = 0.65$) and the EC $\times$ LI interaction ($P = 0.16$) did not show significant effects. Under the optimal EC condition (EC1), nitrate concentrations in the nutrient solution were lower across all LI levels, ranging from 1181 mg L$^{-1}$ at L1 to 717 mg L$^{-1}$ at L3, with no statistically significant differences among the LI treatments. Conversely, under elevated EC (EC2), nutrient solution nitrate concentrations were significantly higher, increasing from 2398 mg L$^{-1}$ at L1 to 2712 mg L$^{-1}$ at L3. All EC2 treatments resulted in significantly greater nitrate levels in the nutrient solution compared to their respective EC1 counterparts (Fig.4b).Leaf nitrate (NO_3_^–^-N) concentration did not show a statistically significant response to EC ($P = 0.198$), LI ($P = 0.641$), or the EC $\times$ LI interaction ($P = 0.617$) (Fig.4c).

**Fig. 5:**
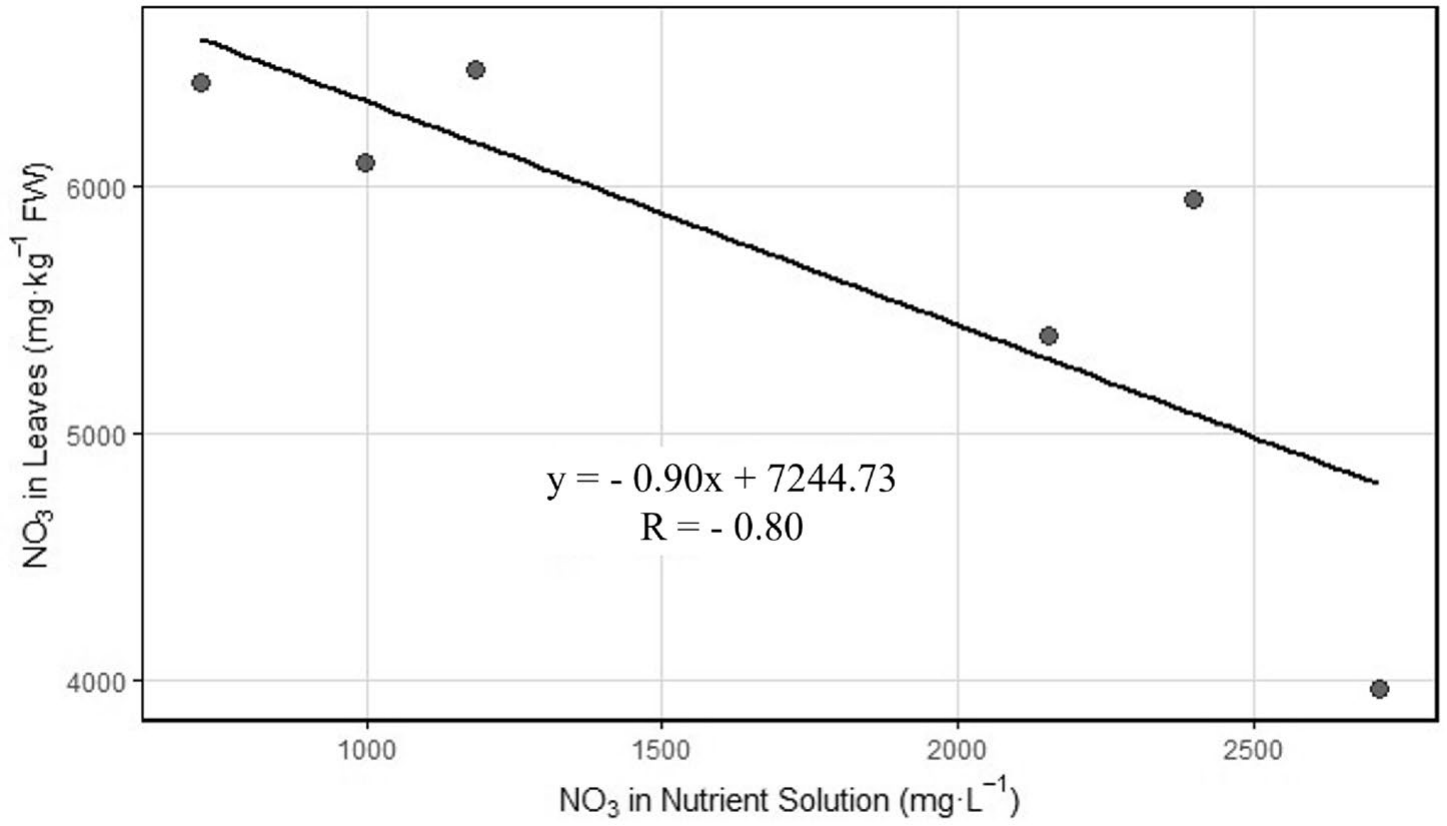
Scatter plot showing a negative linear relationship between solution nitrate concentration and leaf nitrate accumulation across treatments.

The scatter plot (Fig.5) illustrates the relationship between nitrate concentration in the nutrient solution and nitrate accumulation in lettuce leaves across all treatment combinations. Each point represents a unique EC and LI combination. A negative linear relationship was observed, suggesting that higher nitrate availability in the nutrient solution did not translate to higher nitrate accumulation per unit production in the plant tissue. Instead, as solution nitrate levels increased, a decrease in leaf nitrate content occurred, as described by the regression equation $y = -0.90x + 7245$. This trend underscores the physiological inhibition of nitrate uptake or assimilation under high salinity stress.

### Effect of LI and EC on mineral content

Tables3 and 4 show the effect of EC and LI treatments on the composition of macro and micro nutrients in the leaf tissues. The impact of EC on the mineral composition of lettuce leaves was found to influence the uptake of all macronutrients and most micronutrients (except Zn and Mn), with a statistically significant difference. Optimal EC conditions (EC1) consistently resulted in greater concentrations of N, P, K, S, Ca, Mg, Fe, and Cu compared to high EC (EC2) levels ($P < 0.001$). In contrast, LI had no significant effect on the uptake of any mineral (macro and micro) for both groups of minerals. Also, no significant interaction between EC and LI was detected for most nutrients ($P> 0.05$). An exception was observed for B, where an interaction impact of EC $\times$ LI was statistically significant ($P = 0.036$), although no consistent trend was evident across light levels. Zn and Mn concentrations did not differ significantly between EC levels or light treatments (Table 4).

**Table 3:** Effect of EC and LI on macronutrient uptake in lettuce leaf tissue^1^.

**EC**	**LI**	**N (%)**	**P (%)**	**K (%)**	**Ca (%)**	**Mg (%)**	**S (ppm)**
EC1	L1	4.60±0.30	0.90±0.10	6.30±1.60	1.60±0.00	0.50±0.00	2345±145
	L2	4.50±0.30	1.05±0.05	7.65±0.55	1.85±0.05	0.60±0.00	2485±285
	L3	4.45±0.35	0.95±0.05	7.10±0.10	1.80±0.10	0.55±0.05	2355±295
EC2	L1	3.95±0.15	0.70±0.10	4.50±0.90	1.35±0.15	0.40±0.10	1095±325
	L2	3.80±0.20	0.55±0.05	4.20±0.80	1.35±0.15	0.35±0.05	1035±345
	L3	3.30±0.00	0.45±0.05	3.05±0.45	1.15±0.15	0.30±0.10	680±130
EC mean	EC1	4.52±0.14^a^	0.97±0.04^a^	7.02±0.50^a^	1.75±0.05^a^	0.55±0.02^a^	2395±115^a^
	EC2	3.68±0.14^b^	0.57±0.05^b^	3.92±0.43^b^	1.28±0.07^b^	0.35±0.04^b^	936±151^b^
LI mean	L1	4.28±0.23	0.80±0.08	5.40±0.91	1.48±0.09	0.45±0.05	1720±388
	L2	4.15±0.25	0.80±0.15	5.93±1.07	1.60±0.15	0.47±0.07	1760±456
	L3	3.88±0.36	0.70±0.15	5.07±1.18	1.48±0.20	0.43±0.08	1517±501
P-value interaction		0.568	0.125	0.455	0.290	0.455	0.741
P value EC		<0.001	<0.001	<0.001	<0.001	<0.001	<0.001
P value LI		0.617	0.822	0.852	0.814	0.887	0.921

^1^Values represent the means of two statistical replicates per light intensity and EC treatment, with each replicate consisting of six plants. Data were analyzed using a two-way ANOVA. When interaction effects were not significant, a one-way ANOVA was performed. Mean separations were conducted using the least significant difference (LSD) test at$P \le 0.05$. Different letters indicate significant differences among EC means, and letters are shown only when differences were statistically significant. The absence of letters indicates that no significant differences were detected among treatments.

**Table 4:** Effect of EC and LI on micronutrient uptake in lettuce leaf tissue^1^.

**EC**	**LI**	**B (ppm)**	**Zn (ppm)**	**Mn (ppm)**	**Fe (ppm)**	**Cu (ppm)**
EC1	L1	28.05±2.15^a^	17.60±8.20	39.90±17.60	70.25±7.95	7.40±0.70
	L2	31.10±0.40^a^	15.00±6.20	40.95±18.75	67.35±5.15	7.35±1.35
	L3	29.15±0.35^a^	13.70±4.80	38.20±18.50	60.10±4.00	6.65±1.15
EC2	L1	23.40±0.40^b^	17.25±2.05	48.60±14.90	45.20±10.80	3.35±0.85
	L2	19.90±0.70^c^	19.50±3.50	56.30±20.70	40.85±4.45	3.40±1.30
	L3	19.70±0.30^c^	16.25±0.25	43.00±10.30	26.70±4.30	2.20±0.00
EC mean	EC1	29.43±0.80^a^	15.43±3.01	39.68±8.19	65.90±3.27^a^	7.13±0.52^a^
	EC2	21.00±0.79^b^	17.67±1.21	49.30±7.50	37.58±4.77^b^	2.98±0.47^b^
LI mean	L1	25.73±1.61	17.43±3.45	44.25±9.74	57.73±9.07	5.38±1.25
	L2	25.50±3.25	17.25±3.18	48.62±12.23	54.10±8.14	5.38±1.37
	L3	24.42±2.73	14.97±2.09	40.60±8.75	43.40±9.93	4.43±1.36
P-value interaction		0.036	0.886	0.953	0.802	0.966
P value EC		<0.001	0.500	0.400	<0.001	<0.001
P value LI		0.932	0.813	0.862	0.534	0.846

^1^Values represent the means of two statistical replicates per light intensity and EC treatment, with each replicate consisting of six plants. Data were analyzed using a two-way ANOVA. When interaction effects were not significant, a one-way ANOVA was performed. Mean separations were conducted using the least significant difference (LSD) test at$P \le 0.05$. Different letters indicate significant differences among EC means, and letters are shown only when differences were statistically significant. The absence of letters indicates that no significant differences were detected among treatments.

## Discussion

### EC overrides the benefits of LI on growth and yield

Lettuce morphological development responded to both EC and LI in this study, with EC having a more pronounced effect. Across all morphological and biomass-related parameters, plants grown under EC1 (1.5–2.0 dS m^−1^) performed better than those grown under EC2 (4.5–6.0 dS m^−1^). EC2 treatments caused substantial reductions in fresh and dry biomass, canopy expansion, and root growth. In this study, leaf area under EC1L3 was 77% greater than under EC2L3, indicating a pronounced loss of light-intercepting surface and photosynthetically active tissue under elevated EC conditions. Similar reductions in leaf area under saline or high-EC conditions have been widely reported in lettuce and other leafy greens, supporting the sensitivity of canopy development to root-zone salinity. The observed decline in growth under EC2 can be attributed to osmotic stress and ion toxicity, which impair water uptake and restrict cell expansion. Elevated EC increases the osmotic potential of the root-zone solution, reducing water availability, cell turgor, and cell enlargement, while excessive ion accumulation disrupts membrane integrity and ion homeostasis. Together, these processes constrain photosynthetic capacity and biomass accumulation^40,41^. In agreement with our findings^42^ reported that salinity levels exceeding 2.0 and 2.6 dS m^−1^ negatively affected fresh yield and overall plant growth in lettuce, respectively. Root growth closely mirrored shoot responses in our study, indicating that elevated EC restricts not only shoot expansion but also root elongation. Similar root growth limitations under saline conditions have been reported previously and are commonly associated with reduced nutrient acquisition capacity^43–45^. These responses are largely driven by ion imbalances in the root zone and reduced cellular turgor, which together inhibit root cell division and elongation. In addition, the lower nutrient solution pH under EC2 in our study may have further reduced the availability of phosphorus, magnesium, and micronutrients, contributing to the lower tissue concentrations observed. Such nutrient imbalances, combined with the energetic costs associated with osmotic adjustment, likely explain the pronounced growth suppression under EC2 conditions. Consistent with our results, previous studies have shown that high salinity reduces root surface area by limiting root hair development, thereby restricting nutrient absorption^46^. In lettuce, reductions in root size of up to 42% have been reported under NaCl-induced salinity stress^47^. These structural limitations, together with ion channel disruption and membrane depolarization, compromise the uptake of essential nutrients such as K^+^, Mg^2+^, and Ca^2+^^46^, reinforcing the physiological basis for the reduced growth observed in this study.

LI is a key environmental factor regulating plant growth, morphology, and biomass accumulation. Higher light intensities generally promote photosynthesis and carbon assimilation, leading to increased shoot biomass and improved crop productivity. Several studies have highlighted the significant effect of increasing photosynthetic photon flux density (PPFD) or daily light integral (DLI) on lettuce growth and yield. For instance^48^, observed increased lettuce dry biomass and photosynthesis when PPFD was elevated from 125 to 375 $\mu$mol m^−2^ s^−1^in climate-controlled growth chambers, indicating enhanced production under optimal lighting. However, these benefits are not universal; under high salinity or in combination with other environmental stressors, the positive effects of increased LI may be diminished or even reversed^49^. identified that an increase in DLI from 8.6 to 11.5 mol m^−2^ d^−1^ enhanced FW of iceberg lettuce under vertical hydroponic culture, but further increase to 14.4 mol m^−2^ d^−1^had a detrimental effect on biomass, indicating a threshold beyond which increased light becomes harmful. Interestingly^21^, demonstrated that the maximum PPFD for biomass production in lettuce is temperature-dependent, with 500–600 $\mu$mol m^−2^ s^−1^ being optimal at moderate temperatures but potentially stressful at low temperatures. This suggests that high light intensities must be carefully managed relative to other environmental parameters to avoid photoinhibition or nutrient stress. In the present study, the expected benefits of LI were observed under optimal EC (EC1), where increasing LI significantly enhanced FW and morphological traits of lettuce plants. While increased LI is generally associated with enhanced plant growth, the interaction with other factors, such as elevated EC, can abolish such benefits. Indeed, in our study, the growth-promoting effects of LI were no longer evident under the elevated EC (EC2) tested, indicating that high salinity stress can override the beneficial influence of increased light availability.

This outcome of the interactive impact of LI and EC can be further reflected in the light use efficiency (LUE) calculated as in^50^. Under optimal EC1 conditions, LUE declined linearly with increasing LI (from 0.0145 g DW/µmol at L1 to 0.0120 g DW/µmol at L3), indicating diminishing returns at higher PPFD, but remained significantly higher overall compared to EC2. In contrast, under EC2, LUE was drastically reduced across all light levels (ranging from 0.0072 at L1 to 0.0055 g DW/µmol at L3), suggesting that high salinity limits the plant’s capacity to convert light energy into biomass regardless of light input.

These results highlight the cumulative effects of environmental stressors in controlled environments, emphasizing the need for precise nutrient and salinity management to ensure that improvements in lighting strategies translate into actual gains in productivity. They also underscore the critical importance of managing EC in recirculating hydroponic systems to avoid excessive salt accumulation and salinity-induced stress and yield loss.

### Non-interactive influence of EC and LI on nutrient absorption dynamics

EC plays a critical role in regulating mineral nutrient absorption in lettuce. In our study, elevated EC (EC2) consistently reduced leaf nutrient concentrations compared to the optimal EC (EC1), independent of light intensity. These results align with previous research showing that high EC induces osmotic stress and ion competition, which compromise root membrane selectivity and restrict the uptake of key nutrients such as K^+^, Ca^2+^, and NO_3_^–^^51–53^. Mechanistically, salinity stress damages nutrient uptake through physical and physiological processes, including decreased root surface area, membrane depolarization, and down-regulation of specific ion transporters^51^. These responses result in nutrient imbalances even in ion-rich solutions, emphasizing that nutrient availability in the solution does not always equate to successful uptake when salinity exceeds the plant’s tolerance threshold. In accordance with the literature, tested LI did not significantly impact the nutrient content of leaves^5,54^,. Although high light intensity theoretically increases transpiration and nutrient demand, these effects are suppressed under salinity stress or limited nutrient availability. Interestingly, only B exhibited a significant EC $\times$ LI interaction (P = 0.036), likely due to its dependence on transpiration for mobility and susceptibility to environmental variation^55^. A notable and counterintuitive finding was the negative correlation between external nutrient levels and internal nutrient accumulation, supporting the idea of physiological saturation or stress-induced downregulation of transport pathways^54^.

These findings support the hypothesis that nutrient solution management should prioritize ionic balance, osmotic conditions, and plant–environment interactions over simply increasing nutrient concentrations. In hydroponic systems, where precise control is possible, optimizing EC within the plant’s tolerance range is more effective for maintaining consistent nutrient uptake and ensuring crop quality than supplying excessive nutrients.

### Decoupling of external nitrate availability and internal accumulation under elevated EC conditions

Our initial hypothesis was that increased EC of the hydroponic solution would increase nitrate accumulation in lettuce leaves. From the results obtained, leaf nitrate in all samples measured was far below (maximum 321 mg/kg FW, considering 5% dry matter fraction) the maximum allowed nitrate level in lettuce set by the European Commission (5000 mg/kg FW)^56^. Nevertheless, a negative correlation was observed between nitrate levels in the nutrient solution and nitrate levels in leaf tissue (Fig.5). The results show low leaf nitrate concentrations in treatments at high EC (EC2) compared to optimum EC (EC1). Such a negative correlation contradicts classical expectations of nutrient availability and uptake and refers to a salinity-stress-mediated physiological control mechanism. Indeed, regression analysis revealed the presence of a negative linear relation between solution and leaf nitrate concentrations ($y = -0.90x + 7245$; $R =- 0.80$), though the relationship was not significant ($P> 0.05$). These results suggest that excess external nitrate supply does not necessarily promote nitrate accumulation in plants and may even inhibit uptake or assimilation. Under elevated EC, nitrate uptake and assimilation are likely constrained by reduced root activity and salinity-induced inhibition of nitrate reductase activity, limiting the conversion of nitrate into organic nitrogen despite high external availability. A likely explanation is feedback inhibition of nitrate uptake as proposed by^57^ and supported by more recent studies^11,58^. At the biochemical level, this response may result from inhibition of nitrate reductase (NR), the key enzyme responsible for reducing nitrate (NO$_3^-$) to nitrite (NO$_2^-$) during assimilation. Several studies have shown that high salinity impairs NR activity, reducing nitrate reduction even under high external nitrate availability^17,59^. In parallel, salinity can disrupt ion homeostasis, damage root membranes, and reduce energy availability for active transport, thereby further limiting nitrate uptake^51^.

Although EC $\times$ LI interaction was not significant for nitrate concentration, there was a trend towards lower leaf nitrate at elevated light intensity under EC2, consistent with literature showing that greater light stimulates NR activity and enables more reduction of nitrate^60^. This is a widely documented phenomenon, especially under controlled environments, where low light often leads to nitrate accumulation in leafy vegetables^53,61^. Our LI range (145–240 $\mu$mol m^−2^ s^−1^), while moderate, may have been insufficient to fully activate light-dependent nitrate assimilation processes. Experimental studies showed E that LI levels >300 $\mu$mol m^−2^ s^−1^ are more effective in suppressing nitrate accumulation in lettuce^52,59^.

These findings demonstrate that greater nitrate availability in solution does not necessarily result in higher leaf accumulation, particularly under salinity stress. Instead, plant nitrate status reflects the dynamic balance between uptake, assimilation, and environmental factors, notably light and salinity. Our results demonstrate the need for a more subtle control of EC and light level in hydroponic systems, especially when nutritional quality and regulatory compliance are being aimed for.

Overall, this study was conducted under controlled environmental conditions with only one cultivar, which may limit the direct extrapolation of the results to other cultivars. Additionally, environmental factors such as temperature and humidity were maintained within a certain range, which may influence plant responses to EC and LI under commercial production conditions.

## Conclusions

This study evaluated the individual and interactive effects of EC and LI on hydroponic lettuce morphology, yield, leaf mineral composition, and nitrate accumulation. The interaction between EC and LI significantly influenced morphology and yield, with the combination of EC1 (1.5–2.0 dS m^−1^) and L3 supporting the optimal plant development, while elevated EC levels (4.5–6.0 dS m^−1^) negatively impacted plant performance, by reducing nutrient uptake and suppressing growth, thereby counteracting the benefits of increased light. Importantly, elevated EC did not translate into greater leaf nitrate accumulation; instead, nitrate status reflected the balance between uptake, assimilation, and environmental conditions, particularly salinity and light. The clear antagonistic effect of EC on lettuce productivity highlights the need for precise nutrient and salinity control in recirculating hydroponic systems. From a management perspective, hydroponic lettuce production should prioritize maintaining nutrient solution EC within 1.5–2.0 dS m^−1^ while applying moderate-to-high light intensity (approximately 240 µmol m^−2^ s^−1^), as increasing light input cannot compensate for growth limitations imposed by elevated salinity. Furthermore, the observed trends stress the importance of integrated environmental management approaches in CEA. Future work should explore broader gradients of EC and LI to better define threshold effects and improve crop performance under varying stressors. Additionally, examining ion-based control for nutrient balancing, rather than relying solely on EC-based control, could provide a more sustainable strategy for hydroponic production.

## Supplementary Information

Supplementary Information.

## Data Availability

The data that support the findings of this study are available from the corresponding author upon reasonable request.
