# Supplementary material for: Interactive effects of electrical conductivity and light intensity on growth, yield, and nutrient dynamics of hydroponic lettuce
Source: Sci Rep. 2026 Mar 24;16:14803. doi: 10.1038/s41598-026-44508-2 (PMC13168658; doi:10.1038/s41598-026-44508-2)
Supplement: Supplementary file 1 — Supplementary Information. [file 41598_2026_44508_MOESM1_ESM.pdf]

Supplemental Data
Interactive Effects of Electrical Conductivity and Light Intensity on Growth, Yield, and Nutrient Dynamics of Hydroponic Lettuce

Nazmin Akter[1], Laura Cammarisano[*1], and Md Shamim Ahamed[†2]

[1]Department of Plant Sciences, University of California, Davis, USA

[2]Department of Biological and Agricultural Engineering, University of California, Davis, USA

Protocols for Leaf Area Measurement using ImageJ

The following are the protocols that was followed to measure the leaf area of the lettuce using the ImagJ software:

- Leaf area was measured using ImageJ software (National Institutes of Health, USA), which is freely available for download.
- After harvest, lettuce leaves were detached and placed flat on a white background (e.g., white paper or cutting board). A ruler was placed in the same plane as the leaves to provide a reference length.

[*]Corresponding author: lcammarisano@ucdavis.edu

[†]Corresponding author: mahamed@ucdavis.edu

- Images were captured using a digital camera under uniform lighting conditions to minimize shadows and ensure clear contrast between leaves and background. Plant identification was recorded for each image.
- Images were imported into ImageJ by selecting *File → Open*.
- The scale was set by selecting the *Straight Line* tool, drawing a line along a known distance on the ruler, and using *Analyze → Set Scale*. The accuracy of the scale was verified by measuring an additional known distance on the ruler.
- Leaf tissue was separated from the background by adjusting color threshold settings (*Image → Adjust → Color Threshold*). Hue, saturation, and brightness were adjusted as needed for each image, and the "Dark background" option was applied when appropriate.
- Individual leaves were selected using the *Wand Tool*. Multiple leaves within the same image were added using the *Shift* key.
- Leaf area measurements were obtained using *Analyze → Measure* (shortcut: Ctrl+M).
- Total leaf area was calculated as the sum of individual leaf areas and expressed in cm^2. Leaf borders were carefully selected to exclude non-leaf tissue.

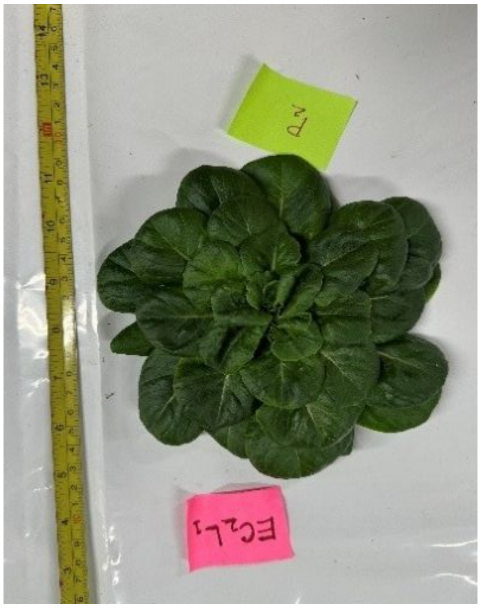

(a) Canopy area

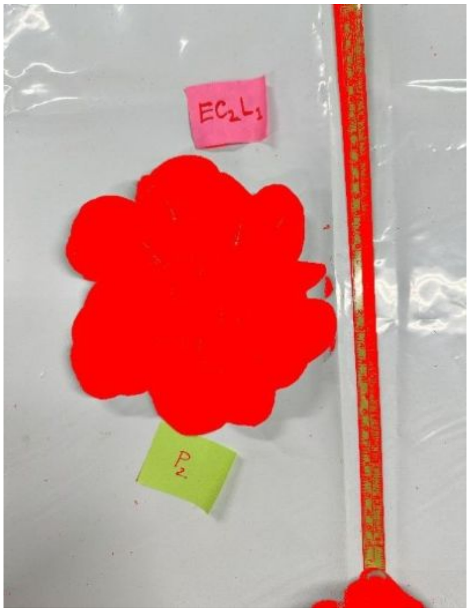

(b) Canopy area image J

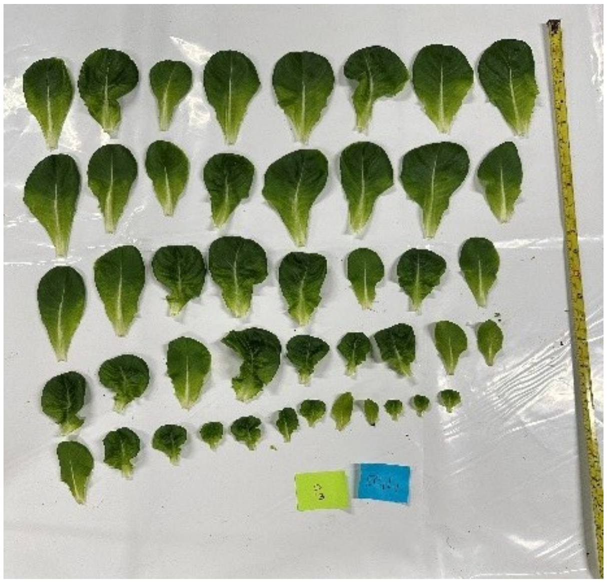

(c) Leaf area

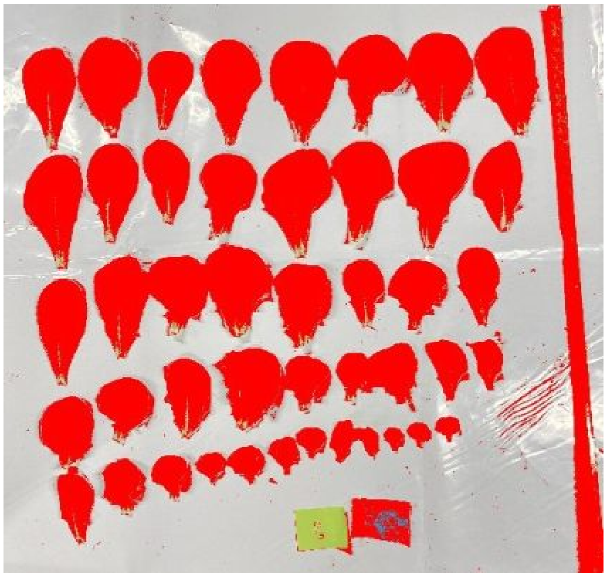

(d) Leaf area image J

Figure S1: Representative images illustrating leaf area measurement using ImageJ. (a,c) Original images of lettuce leaves captured using an iPhone 14 Pro camera with a ruler included for scale. (b, d) Processed images after color threshold adjustment and leaf area selection in ImageJ for canopy diameter and leaf surface area quantification.
